# Supplementary figures and images for: JSTMapp: A web-based joint spatiotemporal modelling and mapping application for epidemiologists
Source: PLoS One. 2025 Dec 2;20(12):e0332607. doi: 10.1371/journal.pone.0332607 (PMC12671773; doi:10.1371/journal.pone.0332607)

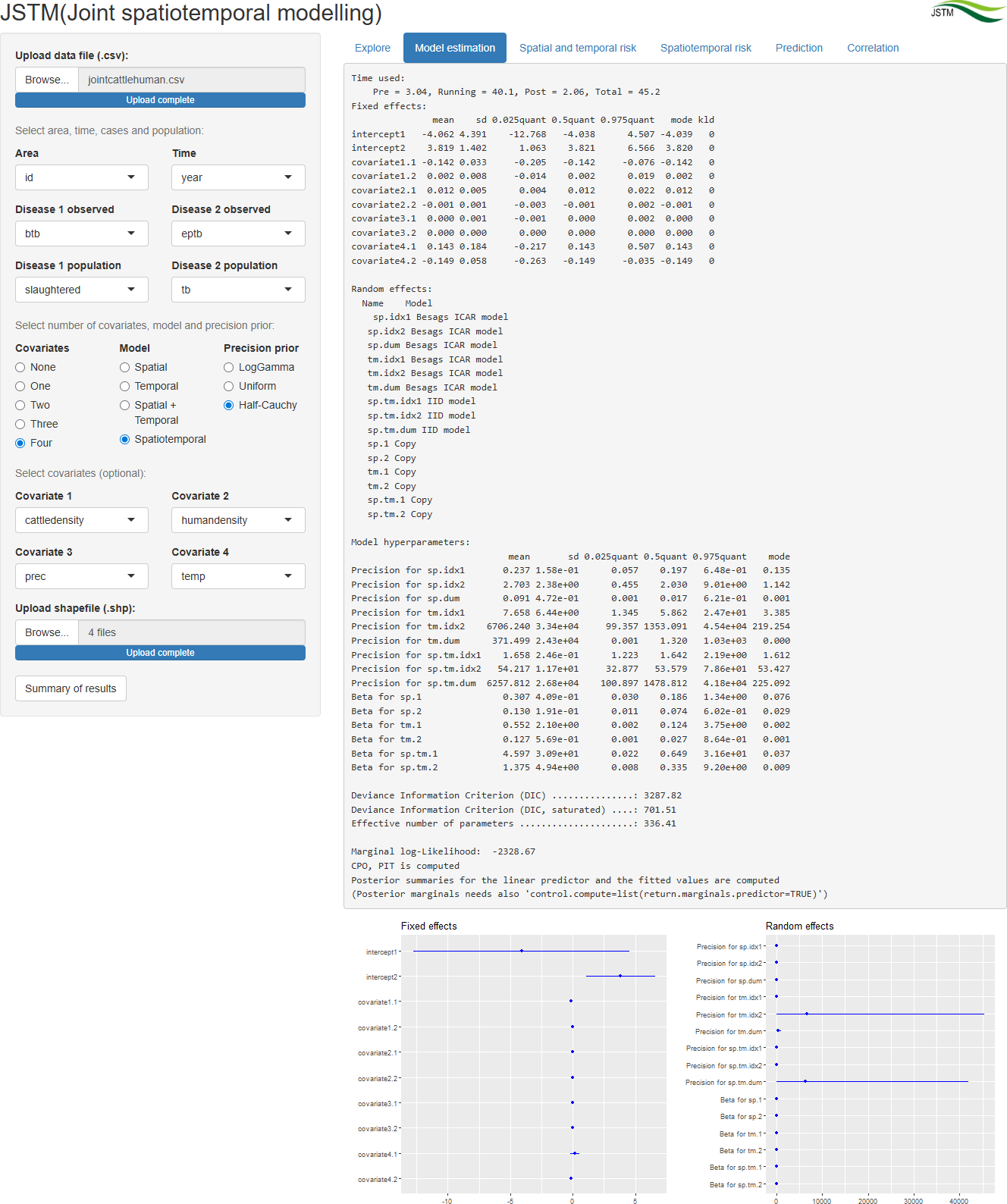

Supplement: S1 Fig — (TIF) [file pone.0332607.s001.tif]
